# Evaluation of the oral ^13^C-bicarbonate technique for measurements of energy expenditure in dogs before and after body weight reduction

**DOI:** 10.1186/s13028-014-0087-6

**Published:** 2014-12-10

**Authors:** Caroline Larsson, Anne Vitger, Rasmus B Jensen, Peter Junghans, Anne-Helene Tauson

**Affiliations:** Department of Veterinary Clinical and Animal Sciences, Faculty of Health and Medical Sciences, University of Copenhagen, Grønnegårdsvej 3, DK-1870 Frederiksberg C, Denmark; Department of Veterinary Clinical and Animal Sciences, Faculty of Health and Medical Sciences, University of Copenhagen, Dyrlægevej 16, DK-1870 Frederiksberg C, Denmark; Leibniz Institute for Farm Animal Biology (FBN), Research Unit Nutritional Physiology “Oskar Kellner”, Wilhelm-Stahl-Allee 2, D-18196 Dummerstorf, Germany

**Keywords:** Energy expenditure, Energy requirements, Obesity, Dogs, Stable isotopes, ^13^C, Tracer technique

## Abstract

**Background:**

Overweight and obesity are the most common nutritional disorders in dogs and may lead to various secondary diseases and decreased lifespan. In obesity research, measurement of energy expenditure (EE) and determination of the energy requirements are essential. The objective with this study was to validate and evaluate the suitability of the oral ^13^C-bicarbonate technique (o^13^CBT) for measuring EE in dog obesity studies. A further objective was to investigate the impact of body weight (BW) reduction and changes in body composition on the EE when measured under conditions corresponding to the basal metabolic rate (BMR).

**Results:**

The EE in five privately owned, overweight dogs was measured simultaneously with the o^13^CBT and indirect calorimetry (IC) for comparison of the results. Two measurements per dog were performed under the same standardised conditions (i.e. fasted and resting state) at the start, and after completing a 12-week BW reduction program. Additionally, measurements of body composition by Dual-energy X-ray absorptiometry (DEXA) were conducted at the beginning and at the end of the BW reduction program. There were no differences in EE results obtained by the o^13^CBT and IC. Overweight and the BW reduction did not affect the estimates for the respiratory quotient (RQ) or the recovery factor for the ^13^C-tracer (RF), both needed when using the o^13^CBT. The dogs lost 16% (SD ± 2.0) of their initial BW in reduced fat mass (*P* < 0.001), whereas fat free mass (FFM) remained unchanged. There was no effect of the BW reduction on the determined EE expressed in kJ/kg BW/d, or in kJ/kg BW^0.75^/d. However, EE was lower (*P* < 0.001) after the BW reduction program when expressed in relation to FFM (kJ/kg FFM/d).

**Conclusions:**

Results from the present study show that the o^13^CBT can be a used in obesity research to determine EE in fasted dogs and under resting conditions. Furthermore, the results suggest that the BMR does not change with reduced BW in overweight dogs as long as the FFM remains unchanged. This indicates that the BMR to maintain one gram of fat is equal to maintaining one gram of FFM in overweight dogs.

## Background

Although several factors may contribute to obesity, such as genetic predisposition, neutering and underlying diseases, it is the life style, i.e. too high dietary intake and lack of physical activity that are primary causes. Obesity is a result of imbalance between energy intake (EI) and energy expenditure (EE) and a sustained increase of the EI not matching the EE, will lead to increased fat deposition and body weight (BW) gain. Previous studies in the United States, Australia, France and the United Kingdom have shown that 34% and up to 59% of the dog populations were overweight or obese [1-4]. Apart from being the most common nutritional disorder in dogs, obesity is often associated with a number of secondary diseases and shortened life span [5]. It is also assumed that overweight and obesity are increasing problems in the dog population [2,5].

Analysis of EE is essential to obesity research. The maintenance energy requirement (MER) depends on the energy required to maintain the basal metabolic rate (BMR), heat increment of the diet, thermoregulation, spontaneous activity and moderate exercise [6]. These factors may vary and lead to large individual variations in the MER. For most moderately active dogs, the BMR, together with the heat increment of the diet are the largest components of their daily MER. The body composition of an animal, usually divided into fat free mass (FFM), fat mass (FM) and bone mineral content (BMC), seems to be the major factor determining the BMR where the FFM is assumed to be the most metabolic active part [7-9]. However, due to variations in the proportions of the metabolically active components making up the FFM, i.e. the skeletal muscles and other organs, individuals with the same body composition might still have different BMR [10].

As many dogs regain BW after a successful BW reduction program, it has been suggested that MER decreases as a result of the BW reduction [11-13], but the knowledge in this field is still very limited. The "golden standard" method for determination of EE is indirect calorimetry (IC), a method based on measurements of respiratory gas exchange (i.e. CO_2_ production and O_2_ consumption). However, apart from respiration units being expensive to build and maintain, this method requires that the dog is confined to a respiration chamber during the measurements, and not all dogs are easily adapted to lie relaxed in the chamber. Alternatively, stable isotope techniques, like the ^13^C-bicarbonate technique (^13^CBT), can be used to estimate the EE in animal and human individuals [14-20]. The advantage with the ^13^CBT is that this method can be used minimally invasive, i.e. by using oral administration of the ^13^C-tracer [18,20] and collection of breath samples. In addition, if the breath samples are collected into breath bags [18,19,21], the method can be used to estimate EE in a more natural environment, or in the veterinary clinic. However, when using the ^13^CBT, appropriate estimates are needed for the respiratory quotient (RQ) and the recovery factor (RF) of the ^13^C-tracer. These important estimates can be found by validating the o^13^CBT against IC, as previously done for lean dogs in the fasted state and during resting conditions [20].

The objective of this study was to evaluate the suitability of the oral ^13^C-bicarbonate technique (o^13^CBT) for obesity studies in dogs and to investigate if BW reduction affects the estimates for RQ and RF. A further objective was to investigate the impact of BW reduction and changes in body composition on the BMR.

## Methods

The experimental procedures followed the Danish National Legislation and guidelines approved by the Member States of the Council of Europe for the protection of vertebrate animals [22], and the experiment was performed at the Faculty of Health and Medical Sciences, University of Copenhagen, Denmark.

### Animals

Five privately owned female dogs were included in the present study (Table 1). All dogs were overweight, with body conditions score (BCS) ≥ 7 on a 9-point scale system where 1 corresponds to emaciated, and 9 to grossly obese [23]. All dogs were scored by the same two observers, both experienced veterinary surgeons. The dogs were allocated to a 12-week BW reduction program at the University Hospital for Companion Animals, University of Copenhagen, Denmark, and BCS was determined at the start and at the end of this program.

**Table 1:** **Dogs included in the study**

				**BW (kg)**			**BCS**	
**Dog**	**Breed**	**Sex**	**Age (yr)**	**Start**	**End**	**Target**	**Start**	**End**
1.	Australian shepherd	FS	3	27.2	23.9	23	7	7
2.	Labrador retriever	FS	4	34.1	29.0	28	7	5
3.	Labrador retriever	F	8	35.0	30.0	29	7	6
4.	Australian shepherd	FS	10	26.7	22.3	22	7	6
5.	Border collie	F	11	26.0	22.5	19	9	8

Breed, sex, age, body weights (BW) and body condition scores (BCS) at the start and at the end of the BW reduction program, and the estimated target BW of the five dogs. FS = Female spayed, F = Female.

During the BW reduction program all dogs were fed the same diet (Royal Canin Satiety Support, Royal Canin, Aimargues, France) rationed to about 260 kJ ME/kg BW^0.75^/d for the estimated target BW (see Calculations). The amount of feed was adjusted every second week if not a BW reduction of 1-2% per week was achieved.

All but one dog (dog 2), were exercised at the hospital three days a week during the BW reduction program. Sessions lasted for one hour, using a combination of underwater treadmill (Hydro Physio HP200, Shor-Line, Kansas City, USA) and dry treadmill (Ultimate Fit Fur Life prototype, Fit Fur Life Ltd., Haslemere, UK) exercise. Dog 2 was exercised by daily walks (~1.5 h) with its owner. All dogs remained healthy throughout the study.

### Measurement of energy expenditure

The dogs were fasted overnight before measurement of EE, and they had free access to water during the time for measurements. Before measurement started, the dogs were weighed and taken for a short walk on a leash. Thereafter, the dogs were placed in a respiration chamber as described in a previous validation study of the o^13^CBT in dogs [20]. Energy expenditure was estimated from simultaneous measurements with the o^13^CBT and with IC. For measurement with the o^13^CBT, samples of breath air were drawn from the respiration chamber every third minutes and analysed online for ^13^C/^12^C ratios by means of an infrared ^13^C isotope analyser (IRIS, Wagner Analysentechnik, Bremen, Germany). After a baseline value was recorded, a dose (5 mg/kg BW) of ^13^C labelled sodium bicarbonate (NaH^13^CO_3_, 98% atom% ^13^C, Sigma-Aldrich, St Louis, MO, USA) mixed with a small amount (~20 g) of liver paté was given orally to the dog. Continuous online IC measurements of the respiratory gas exchange (O_2_ consumption (*R*O_2_) and CO_2_ production (*R*CO_2_)) started immediately after tracer administration. Measurements with both IC and the o^13^CBT lasted for about 4–5.5 h, depending on the behaviour of the dog and the time for the ^13^C/^12^C ratios to return to baseline level. The behavior of the dogs was observed by the same person during all measurements. If a dog was considered not resting, i.e. if it started to become restless or uncomfortable by showing signs such as barking incessantly or moving around more than changing position for resting, the dog was taken out of the chamber. The principles and procedures used for calibration and gas exchange measurements in the respiration chamber have been described previously [24], as well as the detailed procedure used for the o^13^CBT [20].

### Heart rate

Heart rate (HR) was recorded during the time in the respiration chamber using a human heart rate monitor and sensor belt (Polar RS800G3, Kempele, Finland). The sensor belt was attached around the thorax and was further held in place by an elastic bandage. The monitor was attached to the collar.

### Measurement of body composition

At the start and the end of the study, BMC, FM, and FFM were determined by Dual-energy X-ray absorptiometry (DEXA), using a Lunar Prodigy whole-body fanbeam scanner (GE Healthcare, Freiburg, Germany). The dogs were anaesthetized with a premedication of methadone (0.3 mg/kg BW, Comfortan Vet, Dechra Veterinary Products, United Kingdom) in combination with acepromazin (0.015 mg/kg BW, Plegicil Vet, Dechra Veterinary Products, United Kingdom) or diazepam (0.25 mg/kg BW, Stesolid, Actavis, Iceland), followed by intravenous propofol (4 mg/kg BW, PropoVet Multidose, Orion Pharma, Finland) and isoflurane inhalation (Isoflo Vet, Orion Pharma, Finland). The dogs were thereafter scanned in ventral recumbency (head not included). Purpose-designed computer software, validated for humans (enCORE 13.60, GE Healthcare, Freiburg, Germany), was used for data analysis.

It was sought to perform the measurements of EE and measurements of body composition as close in time as possible, which in most cases were within two weeks. Dog 1 and dog 4, however, had the last respiration measurements taken about four weeks after the DEXA-scans and the termination of the supervised treadmill exercise. In this period, these two dogs were exercised by jogging 3–5 km with their mutual owner 3–4 times per week, in addition to daily walks of about 1.5 hours.

### Calculations

#### Estimation of target BW

The target BW for the dogs was estimated from their start BW and BCS. A BCS of 7 (dog 1–4) corresponded to an overweight of approximately 20%, and target BW was calculated as: BW (kg)/1.2. A BCS of 9 (dog 5) corresponded to an overweight of approximately 40% and the target BW was calculated by dividing the start BW (kg) with 1.4 (Table 1).

#### Estimation of EE from IC measurements

EE (kJ/d) was estimated from the measured *R*CO_2_ (l/d) and *R*O_2_ (l/d), using the following equation [25]:

1 $$ \mathrm{E}\mathrm{E}=5.02\cdot R{\mathrm{CO}}_2+16.18 \cdot R{\mathrm{O}}_2-5.9\cdot {N}_u $$

Urine was not collected, because the contribution of the urinary nitrogen excretion (*N*_u_) to the value of EE is usually smaller than 1% [26]. Therefore, the last term of Eq. (1) was neglected.

#### Estimation of EE by using the o^13^CBT

The ^13^C enrichment (ppm) of expired CO_2_ was calculated on the basis of the results from the IRIS. The area under the ^13^C enrichment-time curve (AUC, (ppm ^.^ min)) was determined by using the compartmental modelling module in the SAAM II software [20,27]. Generally, the complete dose of ^13^C administrated (D, (mol)) will not be recovered in the expired air and therefore also an estimate for the RF is needed. The RF was calculated from the CO_2_ production measured with IC and data obtained from the o^13^CBT according to the following equation:

2 $$ \mathrm{R}\mathrm{F}=\frac{R{\mathrm{CO}}_{2,IC}}{\left(\mathrm{D}/\mathrm{A}\mathrm{U}\mathrm{C}\right)} $$

By using the AUC, the tracer dose administrated and the estimated RF, the *R*CO_2,13C_ was calculated according to the following equation [28]:

3 $$ R{\mathrm{CO}}_{2,13\mathrm{C}}=\left(\mathrm{D}/\mathrm{A}\mathrm{U}\mathrm{C}\right)\cdot \mathrm{R}\mathrm{F} $$

EE (kJ/d) was estimated using the same equation (Eq.1) as from IC measurements [25], but since the o^13^CBT only provides an estimate for the *R*CO_2_ and not the *R*O_2_, an estimate for the RQ is needed (Eq.4). The individual RQ´s were obtained by IC measurements (RQ = *R*CO_2_/*R*O_2_) and the mean values from measurements before and after BW reduction were used:

4 $$ {\mathrm{EE}}_{13\mathrm{C}}=5.02\cdot R{\mathrm{CO}}_{2,\ 13\mathrm{C}}+16.18\frac{R{\mathrm{CO}}_{2,\ 13\mathrm{C}}}{\mathrm{RQ}} $$

Results were standardized to 24 h, and the estimated EE (kJ/d) was further calculated in relation to BW (kJ/kg BW/d), metabolic BW (kJ/kg BW^0.75^/d), and FFM (kJ/kg FFM/d).

#### Heart rate

From the recordings taken when the dogs were in the chambers, mean values for HR, expressed in beats per minute (bpm) were calculated.

### Statistical analysis

**Figure 1:**
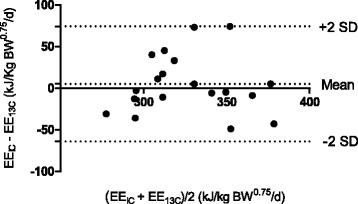
**Bland-Altman plot of determined EE values.** The differences between the paired measurements (EE estimated from measurements with indirect calorimetry (EE_IC_) and with the o^13^CBT (EE_13C_) are plotted against the averages of the two data values. The 95% confidence interval (mean ± 2 SD: 5.1 ± 69.2) is the expected range of the difference between measurements in which 95% of the tests will lie.

Data from the measurements were statistically analysed using the MIXED procedure in SAS® (SAS® Version 9.3, SAS Institute Inc. Cary, North Carolina, USA). The effect of BW reduction on the measured parameters: HR, *R*O_2_, RF, RQ, BW, FM, FFM, were analysed with time, i.e. the start and the end of the BW reduction program, as fixed effect and dog as random effect. The estimated values of *R*CO_2_ and EE determined with IC or the o^13^CBT were tested for difference between the methods, the time for measurements and their interactions as fixed effects, and dog as random effect. The model was then reduced for non-significant interaction effects. Results are presented as least square means (LS-means) with their 95% confidence intervals (95% CI). Effects were considered significant if *P* < 0.05. Additionally, a Bland-Altman analysis was used to compare the EE results determined by IC and the o^13^CBT (Figure 1). The differences between the paired measurements were plotted against the averages of the two data values [29].

## Results

### Body weight, body composition and heart rate

The dogs lost on average 4.8 kg (± 0.5 kg), resulting in 16% decrease of their BW (*P* < 0.001) at the end of the BW reduction program. The FM values were also lower (*P* < 0.01) than the start values for all dogs, whereas FFM remained unchanged (Table 2).

**Table 2:** **Parameters determined by Dual-energy X-ray absorptiometry (DEXA) in dogs (n = 5) allocated to a BW reduction program**

	**Start**	**95% CI**	**End**	**95% CI**	***P*** **-value**
BW	29.8	24.7 - 34.9	25.7	20.6 – 30.8	< 0.001
FFM	14.0	9.7 - 18.3	13.7	9.4 - 18.1	NS
FM	14.2	12.7 - 15.8	10.7	9.2 - 12.3	< 0.01

Body weights (BW), fat free mass (FFM) and fat mass (FM) in kg, determined by DEXA-scans performed at the start and at the end of a 12-week BW reduction program. Results are shown as LS-means with the 95% confidence intervals (CI). NS = not significant.

It was the intention to record HR throughout the measurements. However, for four of the dogs, actual recording time was shorter, since the HR monitor had slipped and lost connection. On two occasions the recording time was reduced to two hours and on two other occasions there were no recordings at all. The average HR did not differ between measurements performed at the start and the end of the BW reduction program (Table 3).

**Table 3:** **Parameters measured in overweight dogs (n = 5) before and after body weight reduction**

	**IC**				**o** ^**13**^ **CBT**				***P*** **-value**	
	**Start**	**95% CI**	**End**	**95% CI**	**Start**	**95% CI**	**End**	**95% CI**	**Method**	**BW reduction**
*R*O_2_, l/kg BW^0.75^/d	16.7	15.4-18.0	15.5	14.3-16.7						NS
*R*CO_2_, l/kg BW^0.75^/d	12.7	11.7-13.7	11.9	10.9-12.9	12.5	11.5-13.4	12.4	11.4-13.4	NS	NS
RF	0.75	0.69-0.82	0.72	0.65-0.78						NS
RQ	0.76	0.71-0.82	0.76	0.72-0.81						NS
HR, bpm	74	65-82	76	68-85						NS
EE, kJ/d	4231	3879-4584	3511	3169-3854	4136	3793-4478	3629	3286-3971	NS	< 0.001
EE, kJ/kg BW/d	145	129-161	140	125-156	142	126-157	147	131-162	NS	NS
EE, kJ/ kg BW^0.75^/d	336	311-362	314	289-338	329	304-354	327	302-352	NS	NS
EE, kJ/ kg FFM/d	314	246-382	263	195-331	309	240-377	275	207-344	NS	< 0.001

Parameters measured in five overweight dogs by indirect calorimetry (IC) and the oral ^13^C-bicarbonate technique (o^13^CBT), two times at the start (n = 10) and two times at the end (n = 10) of a body weight (BW) reduction program. Results are shown as LS-means with the 95% confidence intervals (CI). *R*O_2_ = O_2_ consumption, *R*CO_2_ = CO_2_ production, RF = recovery factor, RQ = respiratory quotient, HR = heart rate, bpm = beats per minute, EE = energy expenditure, FFM = fat free mass, NS = not significant.

### Respiratory quotient and recovery factor

There was no effect of the BW reduction on the estimates of the RF or the RQ (Table 3). The average value for the RF was 0.74 and for the RQ it was 0.76. These values were used as the estimates for RF and RQ in the calculations of *R*CO_2_,_13C_ and EE_13C_, respectively.

### O_2_ consumption, CO_2_ production and energy expenditure

There was no difference between methods on the estimated CO_2_ production or EE. The individual estimated EE values determined by IC and o^13^CBT, plotted in the Bland-Altman plot (Figure 1), were evenly distributed and within the 95% CI.

There was no effect of the BW reduction on the measured *R*O_2_ or *R*CO_2_ (l/kg BW^0.75^/d). The EE in kJ/d was lower (*P* < 0.001) after BW reduction but was not affected after adjustment to BW or BW^0.75^. However, EE expressed in relation to FFM, resulted in lower (*P* < 0.001) values (kJ/kg FFM/d) after BW reduction. The LS-mean values and the 95% CI for the *R*O_2_, *R*CO_2_ and EE are shown in Table 3.

## Discussion

The main objective of this study was to evaluate whether the o^13^CBT may be a useful tool for measurements of EE in BW reduction programs. A further objective was to investigate whether the EE, measured under conditions comparable to BMR, decreases or remains unchanged in dogs after BW reduction. The definition of BMR is the energy required to maintain homeostasis in an animal in a post-absorptive state where the individual is lying down, awake and in a thermoneutral environment to which it has been acclimatized [6]. Prior reported estimates for the BMR in dogs have varied widely with individual values ranging from 200 to 477 kJ/kg BW^0.75^/d [30].

In this study, it could not be controlled if the dogs were sleeping, or moved a little (e.g. sat up and lay down again) during the time in the respiration chamber and this may have affected the results and thereby overestimated their actual BMR. However, if a dog was moving around more than just to change position of rest and showed signs of not be resting calmly or comfortable any longer, the measurement in the chamber was ended. The assumption that the EE measurements were close to BMR in this study is thereby based on the subjective observations of the person outside the chamber and the interpretation of recorded HR data measured while the dogs were inside the chamber. Measurements of HR before and after the time in the chamber would though likely have supported this assumption.

Two methods were used for the estimation of EE, the IC as the reference method and the o^13^CBT. There were no differences between the results obtained by IC or o^13^CBT in this study, confirming the findings of a previous validation study that the o^13^CBT can be used in fasted dogs during resting conditions [20]. However, that study also clearly showed that the estimate for RF may vary depending on factors such as the behaviour of the dog. The RF were significantly higher for dogs being more active (RF = 0.94) during measurements, compared to those being relaxed (RF = 0.74) [20]. The results from the present study indicate that overweight and BW reduction in overweight dogs did not affect the estimate for the RF and an average value of 0.74 was found. This is in agreement with previous studies in fasted dogs [20] and humans [31,32] during resting conditions where RF values between 0.70-0.74 were found after oral administration of the ^13^C tracer. The LS-means means for the RQ was 0.76 at the start as well as at the end of the BW reduction program, which also is similar to what was found previously in fasted dogs [16,20]. Still, the estimates for the RF and RQ need to be determined for each type of standardised measurement situation, i.e. if the estimates are affected of by for example underweight, ageing or ambient temperature.

The dogs in this study were all calm and relaxed in the respiration chamber, which also was reflected in the HR measurements. However, by using breath bags [18,19] and provided that the method is used under standardised conditions, further studies of EE in dog obesity research do not have to be restricted to those dogs that are suitable for measurements in a respiration chamber.

The dogs lost on average 4.8 ± 0.5 kg of their initial BW to the time for the second measurement of body composition. This corresponds to a BW reduction of approximately 16% of their initial BW and is consistent with the expected BW reduction of 1-2% per week. As a result of the BW reduction, the EE (kJ/d) decreased significantly from an average of approximately 4200 kJ/d, to about 3600 kJ/d. However, corrected for metabolic body size, the EE was not affected by the BW reduction, being 335 kJ/kg BW^0.75^/d at the start, and 318 kJ/kg BW^0.75^/d at the end of the BW reduction program.

The conditions for measurement of EE in this study were close to the BMR. For most pet dogs, the BMR represents the major part of their MER. As the BMR (kJ/kg BW^0.75^/d) measured in this study was unaffected by the BW reduction, the results indicate that also the MER is unaffected after 16% BW reduction in obese dogs, provided that the daily physical activity remains unchanged. Physical activity has been suggested to increase the MER by approximately 10% per hour with physical work [33]. This estimated increase can vary greatly depending on factors such as the type and intensity of work and the ambient temperature [33].

The BW reduction program included restricted energy intake combined with regular exercise. The results of measured body composition showed that the FFM remained unchanged, meaning that the BW reduction was a result of reduced FM only. A previous study in overweight dogs suggested that the MER was lower in dogs after BW reduction [13]. Contrary to the present study, the BW reduction in that study was due to a combination of reduced fat mass and lean tissue mass. It is likely that if the BW reduction depends on reduced energy intake alone, the more metabolically active muscle mass will also be reduced. This might lead to a greater decrease in EE (kJ/d) compared to a reduction in FM only and may explain why BMR was affected differently in this study. Calculations of EE in relation to the FFM will give varying results as lean and obese animals may have the same amount of FFM but great variations in FM and EE in kJ/d [7,10,34]. In this study, the EE (kJ/d) decreased and the FFM remained constant after BW reduction, and consequently, the EE expressed as kJ/kg FFM/d was reduced. It is though possible that the time interval between measurements of body composition and EE for two of the dogs at the end of the program might have affected the results when EE was calculated in relation to FFM. During this time, the BW of one of these dogs was further reduced with 1.6 kg. As the exercise on treadmills was terminated with the last measurement of body composition, the muscle mass in these two dogs would likely decrease with time as a result of less exercise. However, in the period between the last measurements of body composition, until the last measurements of EE four weeks later, both dogs were exercised by regular jogging and long walks with their owner. Thus it was assumed that FFM was not reduced in this period and that the additional BW loss of 1.6 kg was attributable to loss of FM.

The presence of FM might stimulate the metabolic rate of other tissues [35]. Thereby the amount of FM will also affect the contribution of FFM to the BMR. Whether the reduced EE (kJ/d) was a result of the decreased FM or due to changes in the more metabolic active FFM, or a combination of both, could not be elucidated in this study. When calculating the EE in relation to kg BW or kg BW^0.75^, the results from this study suggest that the BMR does not change with BW reduction as a result of reduced FM. However, except from one dog, the BCS was still above the ideal at the end of the study and thus, the results indicate that the BMR to maintain one gram of fat is identical to maintaining one gram of FFM in overweight dogs.

The EE-results were based on short-term measurements in the fasted state and in a respiration chamber and factors such as normal daily activities and feeding were excluded. For the o^13^CBT to be useful to provide accurate estimates of the dogs´ MER, all factors influencing the daily EE also have to be included. Further investigations are required to elucidate if the o^13^CBT can be used under other conditions than in fasted and resting state (i.e. in relation to feeding and physical activity). Nevertheless, for dogs being inactive most of the day, the EE measured during fasting and resting conditions may, in a feasible and harmless way, help to improve the guidelines for the MER. Measurement of BMR could be included in BW reduction programs as a routine examination before and after BW reduction to give an indication whether the MER is reduced, and if the BW reduction is a result of reduced FFM or only FM.

## Conclusions

The results from this study show that the minimal invasive oral ^13^C-bicarbonate technique (o^13^CBT) may be a useful tool for further studies in obesity research in dogs. The estimates for the RQ and the RF before and after 16% BW reduction in overweight dogs were not different, provided the measurements were conducted in fasted state and during resting conditions. Energy expenditure (kJ/BW^0.75^/d) measured in dogs under conditions close to the BMR was not affected by BW reduction as a result of reduced FM when the BW still was above ideal. Thus, the results indicate that the BMR to maintain one gram of fat is identical to maintaining one gram of FFM in overweight dogs.

## Abbreviations

AUC: Area under the curve
BCS: Body conditions score
BMR: Basal metabolic rate
BMC: Bone mineral content
BW: Body weight
BW^0.75^: Metabolic body size
CI: Confidence intervals
D: Dose
DEXA: Dual-energy X-ray absorptiometry
EE: Energy expenditure
EI: Energy intake
F: Female
FFM: Fat free mass
FM: Fat mass
FS: Female spayed
MER: Maintenance energy requirement
HR: Heart rate
IC: Indirect calorimetry
LS-means: Least square means
N_u_: Urinary nitrogen
NS: Not significant
o^13^CBT: oral ^13^C-bicarbonate technique
*R*CO_2_: CO_2_ production
*R*O_2_: O_2_ consumption
RF: Recovery factor
RQ: Respiratory quotient
SD: Standard deviation
